# Clinical, Neuroimaging, and Molecular Characterization of a Juvenile PLAN-like Phenotype in Two Siblings Carrying p.Arg645Gln and an Unresolved Exon 4–7 Copy-Number Gain: A Case Report

**DOI:** 10.3390/genes17091132

**Published:** 2026-09-16

**Authors:** Małgorzata Janeczko-Czarnecka, Maciej Gręda, Dorota Cichosz, Robert Śmigiel, Mateusz Biela

**Affiliations:** 1 Department of Pediatric Neurology, T. Marciniak Lower Silesian Specialist Hospital—Emergency Medicine Center, Gen. Augusta Emila Fieldorfa Street 2, 54-049 Wroclaw, Poland; ml.janeczko@gmail.com (M.J.-C.);; 2 Department of Pediatrics, Endocrinology, Diabetology and Metabolic Diseases, Wroclaw Medical University, Chałubińskiego 2a Street, 50-368 Wroclaw, Poland; robert.smigiel@umw.edu.pl

**Keywords:** *PLA2G6*, PLAN, juvenile PLAN, atypical neuroaxonal dystrophy, neurodegeneration with brain iron accumulation, cerebellar atrophy, copy-number variant

## Abstract

PLA2G6-associated neurodegeneration (PLAN) is an autosomal recessive neurodegenerative spectrum encompassing infantile, juvenile/atypical and adult-onset phenotypes. Juvenile PLAN may initially resemble autism spectrum disorder or nonspecific developmental regression, delaying diagnosis. We describe a 14-year-old boy and his 9-year-old sister, both with initially normal early development, followed by progressive gait impairment, speech and cognitive regression, epilepsy or paroxysmal events, contractures and loss of independent ambulation. Brain MRI in both siblings demonstrated marked symmetric cerebellar atrophy, susceptibility-weighted hypointensity of the globus pallidus and substantia nigra compatible with iron accumulation and bilateral optic nerve thinning. Electroneurography showed selective motor axonal involvement. Molecular testing identified the same two heterozygous *PLA2G6* findings in both children: c.1934G>A (p.Arg645Gln), classified as likely pathogenic by the diagnostic laboratory, and a copy-number gain encompassing exons 4–7. Parental testing was unavailable; therefore, the phase of the findings could not be established, and it remains unknown whether they are in trans or in cis. The shared phenotype is highly suggestive of juvenile PLAN. However, unresolved phase and incomplete structural characterization of the copy-number gain preclude confirmation of biallelic *PLA2G6* involvement and definitive molecular attribution. These related cases provide a descriptive account of a juvenile PLAN-like phenotype, without independently attributing the phenotype to p.Arg645Gln, establishing variant pathogenicity or a specific genotype–phenotype correlation.

## 1. Introduction

Neurodegeneration with brain iron accumulation (NBIA) comprises a genetically heterogeneous group of progressive disorders characterized by abnormal iron deposition, most prominently in the globus pallidus and substantia nigra. The estimated prevalence is 1–3 per 1,000,000 [1,2]. Clinical manifestations vary by subtype and may include developmental or cognitive regression, dystonia, spasticity, parkinsonism, ataxia, dysarthria, dysphagia, visual impairment and peripheral neuropathy. Treatment remains predominantly symptomatic [3]. The major genetically defined NBIA disorders include pantothenate kinase-associated neurodegeneration, *PLA2G6*-associated neurodegeneration (PLAN), mitochondrial membrane protein-associated neurodegeneration and beta-propeller protein-associated neurodegeneration [4,5]. Additional forms are associated with variants in *FA2H*, *ATP13A2*, *COASY*, *FTL1*, *CP*, *DCAF17*, *GTPBP2*, *VAC14*, *RAB39B*, and *FTH1* [6,7,8].

*PLA2G6*, located at 22q13.1, encodes calcium-independent phospholipase A2β (iPLA2β), an enzyme involved in phospholipid remodeling, membrane homeostasis, intracellular signaling and the response to oxidative membrane injury [9,10]. Dysfunction of iPLA2β is thought to impair membrane repair and contribute to axonal degeneration. Characteristic MRI findings include cerebellar atrophy, susceptibility-related signal loss in the globus pallidus and substantia nigra and, in some patients, claval hypertrophy or other brainstem abnormalities [10,11,12]. Brain iron accumulation may be subtle or absent, particularly early in the disease course.

PLAN is increasingly regarded as an age-related phenotypic continuum rather than a set of strictly discrete entities. Infantile PLAN (classically termed infantile neuroaxonal dystrophy) usually begins within the first 2–3 years of life and progresses rapidly, with developmental regression, axial hypotonia, spastic tetraparesis, ataxia, optic atrophy and loss of independent ambulation [11,13,14,15,16]. Juvenile PLAN, historically termed atypical neuroaxonal dystrophy (ANAD), generally has a later and more slowly progressive onset, often beginning with gait disturbance, speech regression, cognitive or behavioral change and reduced social interaction. These features may initially lead to a diagnosis of autism spectrum disorder [3,13,17,18]. Adult PLAN most often presents with dystonia–parkinsonism, bradykinesia, cognitive decline and neuropsychiatric symptoms [19,20,21].

To ensure terminological clarity throughout this manuscript, we use the terms ‘juvenile PLAN’ and ‘atypical neuroaxonal dystrophy (ANAD)’ synonymously to denote the childhood-to-adolescence-onset clinical phenotype within the PLAN spectrum, thereby distinguishing it from both the rapid-onset infantile neuroaxonal dystrophy (INAD) and adult-onset dystonia–parkinsonism.

Here, we report two siblings with a juvenile PLAN-like phenotype and the same two heterozygous *PLA2G6* findings: c.1934G>A (p.Arg645Gln) and an exon 4–7 copy-number gain. We focus on the clinical, neuroimaging, electrophysiological and molecular findings, while explicitly addressing the interpretative limitations resulting from unavailable parental segregation studies and unresolved variant phase.

## 2. Case Presentation

### 2.1. Diagnostic Approach

This report was prepared in accordance with the CARE guidelines. Clinical data were reconstructed from available medical records and a structured interview with the children’s mother because documentation from previous care in Ukraine was incomplete. Both siblings underwent pediatric neurological examination, routine laboratory testing, electroencephalography (EEG), electroneurography (ENG) and brain MRI on a 1.5-T scanner without contrast administration including axial T1-weighted, T2-weighted, fluid-attenuated inversion recovery (FLAIR) imaging, diffusion-weighted imaging (DWI), and susceptibility-weighted imaging (SWI), as well as sagittal and coronal T1- and T2-weighted sequences. The MRI images were evaluated by a highly experienced neuroradiologist.

### 2.2. Molecular Genetic Analysis

Genomic DNA libraries were prepared using the Twist Human Core Exome Plus capture kit (Twist Bioscience) and sequenced on an Illumina platform. The laboratory assay therefore used exome-scale capture and next-generation sequencing (NGS), whereas the primary bioinformatic analysis and clinical interpretation of single-nucleotide variants, small insertions/deletions and copy-number variants were restricted to a phenotype-associated virtual panel of approximately 1000 genes associated with rare disorders described in OMIM. The laboratory also performed a limited secondary-findings analysis of known pathogenic ClinVar variants and variants in genes on the ACMG-recommended list. Beyond this limited screening, the remaining exome data were not subjected to comprehensive exome-wide analysis or clinical interpretation. The mitochondrial genome (mtDNA) was not analyzed. No separate analysis of nuclear-encoded genes associated with mitochondrial disorders was documented, and the available report does not specify which of these genes were included in the virtual panel. The mean sequencing depth was approximately 100×, more than 99% of the captured library was covered at ≥30×, and coverage of *PLA2G6* at ≥30× was 100%. Sequence analysis used the GRCh38/hg38 genome assembly and included coding regions and adjacent intronic sequences according to MANE Select transcripts.

The heterozygous *PLA2G6* variant NM_003560.4:c.1934G>A, p.(Arg645Gln) (rs780784951), had a reported population frequency of 0.0013% and was classified as likely pathogenic according to the diagnostic laboratory’s ACMG assessment. Copy-number analysis was derived from the NGS data. The laboratory report did not specify the CNV-calling algorithm. In both siblings, an increased copy-number signal at GRCh38 chr22:38,132,831-38,143,423 (copy-number state 3), encompassing exons 4–7 of *PLA2G6* transcript NM_003560.4, was detected. The report noted correspondence to ClinVar Variation ID 1076982, classified as pathogenic on the basis of a single submission, but did not provide a formal ACMG/ClinGen CNV scoring assessment. No orthogonal confirmation, breakpoint characterization, orientation analysis, or transcript-level study was reported or available to the authors. The report also noted that CNV analysis derived from NGS data cannot establish whether an exon-level copy-number gain represents a tandem duplication and recommended confirmation by a dedicated method such as MLPA. Parental segregation testing was unavailable; therefore, the phase of the two *PLA2G6* findings could not be determined.

### 2.3. Family Overview

The patients were a 14-year-old boy and his 9-year-old sister, born to non-consanguineous parents. Both had been evaluated and treated previously in Ukraine. The available family history included an unspecified neurological disorder in the paternal grandfather. The father had died suddenly before segregation testing could be performed and the mother did not consent to genetic testing. Consequently, the parental origin and cis/trans phase of the two *PLA2G6* findings could not be determined.

### 2.4. Patient 1

Pregnancy and delivery were unremarkable. Early development was age appropriate: the boy sat independently at 6 months, walked at 12 months, and spoke his first words at 12 months. At 3 years of age, he developed nocturnal episodes characterized by sitting up in bed, with open eyes and generalized increased muscle tone. The episodes lasted approximately 30 s and occurred about three times weekly. EEG showed right-sided epileptiform discharges, and carbamazepine was started. According to the mother, seizures ceased, but treatment was discontinued after 3 weeks because of perceived worsening of development. A second antiseizure medication was used for approximately 3 months and was also discontinued for the same reported reason. At 5 years of age, he was diagnosed with autism spectrum disorder. Thereafter, progressive gait instability with frequent falls and speech regression, including loss of sentence formation, became apparent. Brain MRI at 5 years showed cerebellar atrophy and a follow-up study at 7 years was reportedly similar. Achilles tendon lengthening was performed at 11 years. From 12 years of age, the mother observed approximately six daily episodes of generalized limb stiffening, drooling, abnormal breathing, and fixed forward gaze. These episodes could reportedly be interrupted by distraction and their epileptic nature remained unclear. Although an EEG performed during drowsiness and spontaneous sleep showed abnormal spatial organization with suspected right frontal epileptiform activity, the overall clinical presentation was not consistent with definite epileptic seizures. Neurological follow-up was recommended, including video documentation of any concerning events, but no recordings were provided and no further episodes were reported. Given this uncertain picture, initiation of antiseizure medication was not considered clinically indicated. At 14 years, he developed hematemesis and was diagnosed with esophagitis following gastroscopy.

At admission, the boy was nonverbal and produced only isolated inarticulate sounds. Cooperation and eye contact were limited. Head circumference was 54 cm, reported as approximately the 10th percentile according to Polish national growth charts from the Institute of Mother and Child (IMiD). Neurological examination showed mild right convergent strabismus, increased muscle tone that was more pronounced in the upper limbs, bilateral ankle contractures, marked calf muscle atrophy, scoliosis and pes planovalgus. Patellar and left Achilles reflexes were absent, and the right Achilles reflex was weak. He was unable to walk independently and could ambulate only with substantial support with flexed knees. Creatine kinase activity was mildly elevated at 393 U/L (reference range, 39–308 U/L), and lactate was 3.4 mmol/L (reference range, 0.5–2.2 mmol/L).

Molecular testing identified a heterozygous *PLA2G6* c.1934G>A (p.Arg645Gln) missense variant, classified as likely pathogenic by the diagnostic laboratory and an increased copy-number signal at 22q13.1 (GRCh38 chr22:38,132,831-38,143,423; copy-number state 3), interpreted as a heterozygous copy-number gain encompassing exons 4–7 of transcript NM_003560.4. The laboratory report noted that this copy-number gain corresponded to a ClinVar entry classified as pathogenic on the basis of a single submission (Variation ID 1076982); however, no orthogonal validation or structural characterization was available. Because segregation testing was unavailable, the phase of the two findings and compound heterozygosity could not be established.

**Figure 1:**
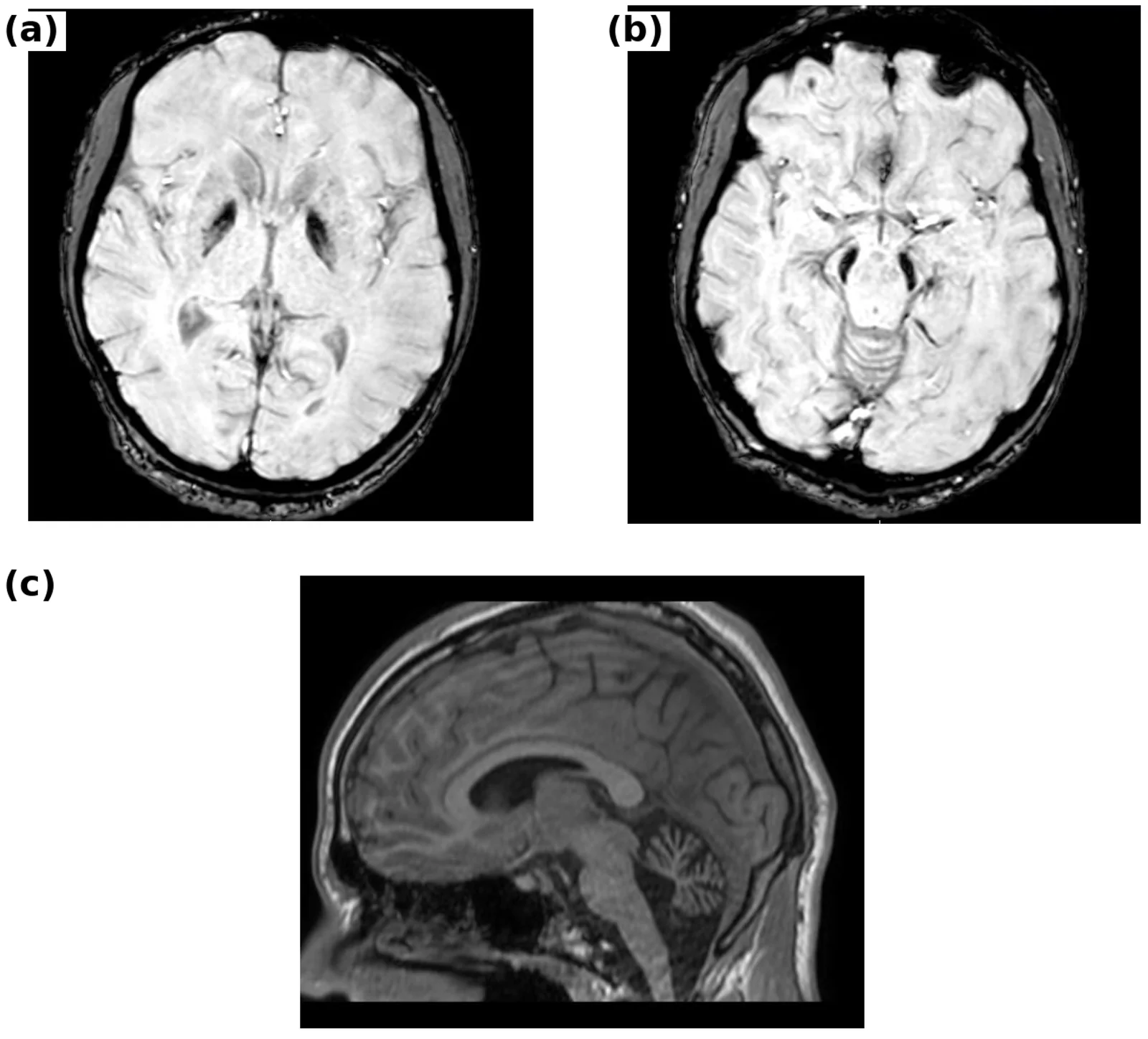
Brain MRI of Patient 1. (**a**) Axial susceptibility-weighted image showing bilateral hypointensity of the globus pallidus. (**b**) Axial susceptibility-weighted image showing hypointensity of the substantia nigra. (**c**) Sagittal T1-weighted image showing marked cerebellar atrophy.

Brain MRI demonstrated marked symmetric cerebellar atrophy, susceptibility-weighted hypointensity of the globus pallidus and substantia nigra compatible with iron accumulation, and moderate bilateral optic nerve thinning (Figure 1). ENG showed reduced motor response amplitudes in both peroneal nerves, consistent with selective motor axonal involvement. Mildly prolonged F-wave latencies were also observed; this finding alone did not establish proximal demyelination or progression. The overall phenotype was considered strongly consistent with juvenile PLAN. The patient was referred for home hospice care and multidisciplinary follow-up, including pediatric neurology, ophthalmology, otolaryngology, orthopedics, pulmonology, rehabilitation, physiotherapy, speech therapy, gastroenterology, psychiatry and psychology.

### 2.5. Patient 2

Pregnancy and delivery were unremarkable. Early development was age appropriate: the girl sat independently at 6 months, walked at 12 months, and spoke her first words at 18 months. Gait deterioration began at approximately 2 years of age, followed by speech regression at 3 years. At the same age, she experienced approximately 10 tonic seizures lasting about 1 min, characterized by hyperventilation, a fall, generalized extension and increased muscle tone, and fixed forward gaze. Valproic acid was initiated. Brain MRI at 4 and 5 years demonstrated cerebellar atrophy. EEG performed approximately 3 years after epilepsy onset showed isolated high-amplitude sharp and slow waves in the left temporal and right frontocentral regions. She remained seizure-free for approximately 3 years. At 6 years of age, the valproic acid dose was reduced following pancreatitis. At admission, she used simple sentences, understood simple commands, and responded to her name. Neurological examination showed increased tone in the upper limbs, absent patellar and Achilles reflexes, bilateral ankle flexion contractures, and a broad-based unsteady gait. She could not walk independently and required hand support. Laboratory testing showed a subtherapeutic serum valproate concentration, low vitamin D concentration, mildly increased aspartate aminotransferase activity, and thrombocytosis; creatine kinase and lactate were within their respective reference ranges. Molecular testing identified the same heterozygous *PLA2G6* findings as in her brother: c.1934G>A (p.Arg645Gln) and an exon 4–7 copy-number gain. Their phase could not be determined.

**Figure 2:**
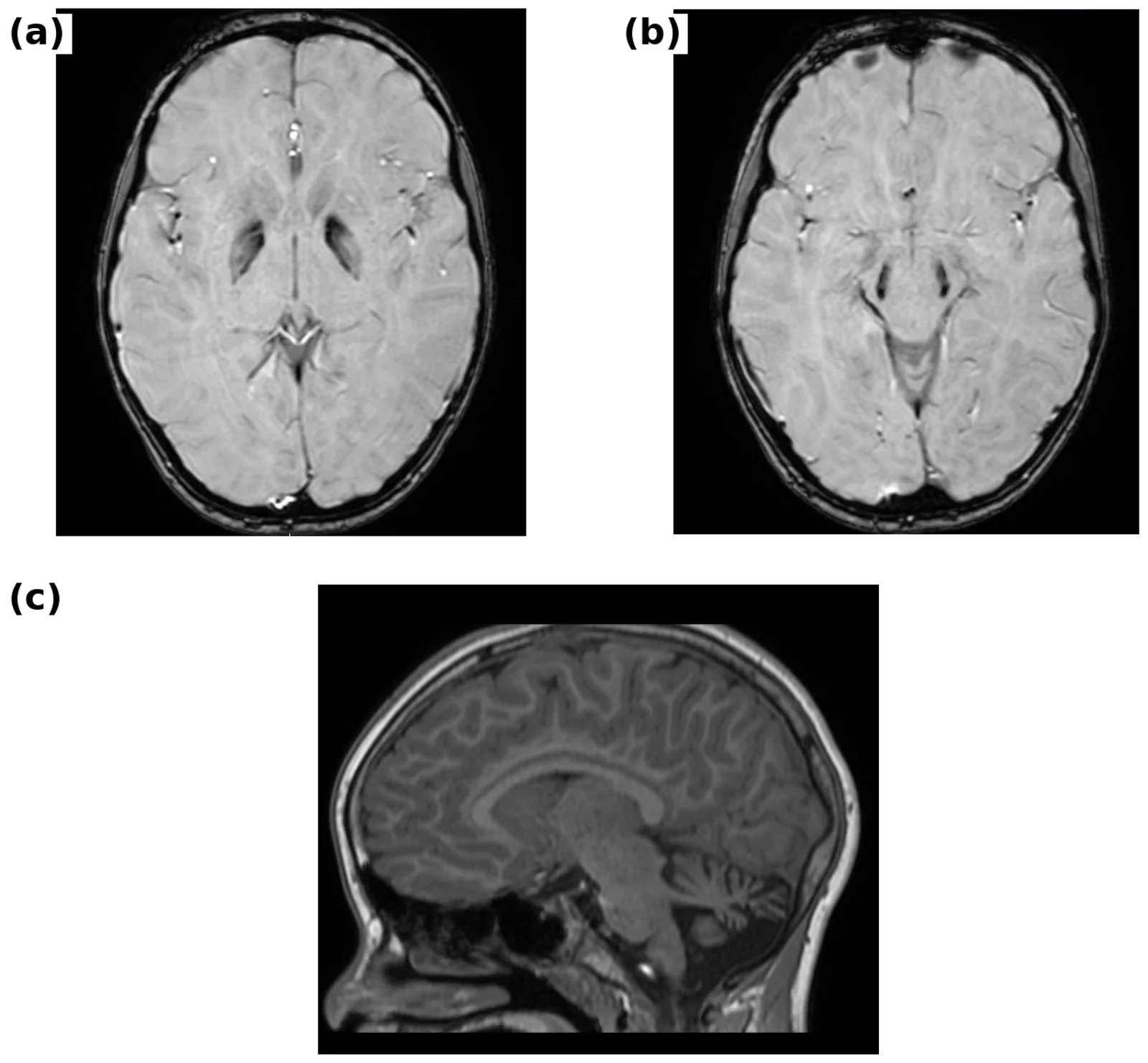
Brain MRI of Patient 2. (**a**) Axial susceptibility-weighted image showing bilateral hypointensity of the globus pallidus. (**b**) Axial susceptibility-weighted image showing hypointensity of the substantia nigra. (**c**) Sagittal T1-weighted image showing marked cerebellar atrophy.

Brain MRI showed marked symmetric cerebellar atrophy, susceptibility-weighted hypointensity of the globus pallidus and substantia nigra compatible with iron accumulation, and moderate bilateral optic nerve thinning (Figure 2). EEG demonstrated groups of theta-frequency slow waves in the anterior regions. ENG showed reduced motor response amplitudes in peroneal nerve fibers supplying the extensor digitorum brevis, with relative sparing of fibers to the tibialis anterior, consistent with mild selective motor axonal involvement. Given an approximately 4-year seizure-free period despite a subtherapeutic valproate concentration, valproic acid was discontinued. The overall phenotype was considered strongly consistent with juvenile PLAN. The patient was referred for home hospice care and follow-up by pediatric neurology, rehabilitation, physiotherapy, speech therapy, and gastroenterology. A comprehensive comparison of the clinical, neuroimaging, electrophysiological, laboratory and genetic findings of both siblings is summarized in Table 1.

## 3. Discussion

The two siblings presented with a highly concordant juvenile neurodegenerative phenotype characterized by initially normal early development, childhood-onset gait impairment, progressive speech and cognitive regression, epilepsy or paroxysmal events, contractures, loss of independent ambulation, cerebellar atrophy, basal ganglia susceptibility changes, optic nerve thinning and motor axonal involvement. This constellation is highly suggestive of juvenile PLAN (historically termed atypical neuroaxonal dystrophy [ANAD]). However, the disorder cannot be considered molecularly confirmed because the phase of the two *PLA2G6* findings remains unresolved and the exon 4–7 copy-number gain has not been structurally characterized.

The age at onset and clinical progression in both siblings resemble previously reported juvenile PLAN (ANAD) cases. Jain et al. described a girl who developed gait difficulty at 3 years, followed by language regression, cognitive decline, spasticity and loss of independent ambulation, with cerebellar atrophy and iron accumulation on MRI [17]. Larger pediatric series similarly identify gait disturbance, developmental regression, cerebellar signs, dystonia or spasticity, and variable epilepsy as common manifestations [9,22,23]. The initial diagnosis of autism spectrum disorder in Patient 1 also reflects a recognized diagnostic pitfall: social withdrawal, reduced communication, and speech regression may precede more overt motor signs in juvenile PLAN [13,18].

Despite their shared molecular findings, the siblings differed in severity. The boy had more profound language loss, severe loss of ambulation, calf atrophy and mildly increased lactate and creatine kinase, whereas his younger sister retained simple speech and limited assisted walking. Intrafamilial phenotypic variability is well recognized across the PLAN spectrum. Pérez-Torre et al. reported markedly different phenotypes in two siblings carrying the same compound heterozygous *PLA2G6* variants, while Blake et al. described two sisters with early regression and dystonia but differences in disease course and complications [24,25]. Age at evaluation may partly explain the differences in our patients, but additional genetic, epigenetic, and environmental modifiers are also plausible.

The mildly elevated creatine kinase (CK; 393 U/L) and lactate (3.4 mmol/L) in Patient 1 are nonspecific findings of uncertain cause and clinical significance. Neither muscle injury related to increased tone nor mitochondrial dysfunction was demonstrated as their cause. No causal relationship with the paroxysmal events of uncertain nature was established. The available ENG findings supported selective motor axonal involvement but did not establish the cause of the biochemical abnormalities or independently exclude a coexisting muscle disorder.

Exome-scale capture and sequencing were performed, but the primary bioinformatic analysis and clinical interpretation were restricted to an approximately 1000-gene virtual panel, including CNV analysis. Apart from the limited secondary-findings screening described in Section 2.2, the remaining exome data were not comprehensively analyzed or interpreted, and the mitochondrial genome was not analyzed. No separate analysis of nuclear-encoded mitochondrial disease genes was documented. These limitations restrict the assessment of alternative or co-occurring genetic diagnoses; the mild lactate elevation alone does not establish a primary mitochondrial disorder.

Neuroimaging provided strong clinical support for a PLAN-like phenotype. Both children had marked cerebellar atrophy and susceptibility-weighted hypointensity of the globus pallidus and substantia nigra. Cerebellar atrophy is often an early and consistent finding, whereas detectable brain iron may appear later or remain absent in some patients [9,12,18,22,23]. In this clinical and radiological context, the bilateral hypointensity of the globus pallidus and substantia nigra is highly suggestive of iron accumulation. SWI is not entirely specific for iron, and quantitative susceptibility mapping (QSM) and R2* relaxometry were unavailable. No specific imaging evidence supported calcification, microhemorrhage, or artefact as an alternative explanation. The bilateral optic nerve thinning is also compatible with PLAN-related optic pathway involvement and complements the optic nerve pallor and ocular motor abnormalities reported in pediatric cohorts [11,22].

The ENG findings were consistent with selective motor axonal involvement. Peripheral neuropathy and hyporeflexia are recognized features of infantile and juvenile PLAN and may coexist with central pyramidal or extrapyramidal signs [3,13,22]. Patient 1 had reduced motor response amplitudes in both peroneal nerves and mildly prolonged F-wave latencies. Patient 2 had reduced peroneal motor response amplitudes recorded from the extensor digitorum brevis, with relative sparing of responses recorded from the tibialis anterior. These cross-sectional observations describe differences between the siblings but cannot establish longitudinal progression, active axonal loss, proximal spread, or successive stages of a length-dependent dying-back process. The mildly prolonged F-wave latencies in Patient 1 are nonspecific and do not establish selective loss of the fastest-conducting fibers or secondary proximal demyelination. The differences may reflect age at assessment, disease severity, or intrafamilial variability, but the available data cannot distinguish among these possibilities [24,25].

The c.1934G>A (p.Arg645Gln) variant should not be described as novel. It was previously reported in two siblings with juvenile PLAN (reported as ANAD) in a French cohort, where it occurred in trans with the truncating p.Tyr790* variant; the authors proposed that p.Arg645Gln might contribute to a comparatively milder juvenile phenotype [23]. The clinical, neuroimaging, and electrophysiological findings in our siblings are broadly concordant with that previously reported spectrum. Because both siblings share the same incompletely characterized molecular findings and their phase remains unresolved, these observations cannot independently attribute the phenotype to p.Arg645Gln, establish variant pathogenicity, or confirm a variant-specific genotype–phenotype correlation. Their concordance with previous reports involving p.Arg645Gln should therefore be interpreted as descriptive rather than confirmatory.

Copy-number variants are a recognized component of the *PLA2G6* mutational spectrum. An exon 4–7 copy-number gain has previously been described in a patient with PLAN and was shown at the transcript level to create an abnormal exon 7–exon 4 junction [18]. In the present siblings, however, the exon 4–7 finding was inferred from an increased copy-number signal and was not characterized at the breakpoint or transcript level. Accordingly, we use the structurally neutral term ‘copy-number gain encompassing exons 4–7’. Orthogonal confirmation of the copy-number change alone would not establish its location, orientation, or transcript-level consequences; these require appropriate structural and, where feasible, RNA analyses.

The principal interpretative limitation is the absence of parental testing. Because both findings were detected in each sibling, they could be in trans and consistent with compound heterozygosity, but they could also be in cis on a single inherited allele. The shared phenotype in two siblings increases clinical suspicion but does not resolve the phase. Maternal testing, if it becomes available, may be informative: identification of only one of the two findings in the mother would support an in-trans configuration in the siblings, whereas detection of both findings would not by itself determine their phase. Direct phasing, long-read sequencing, breakpoint-specific analysis, RNA studies, or testing of another informative relative may therefore still be required depending on the maternal result.

The observed clinical overlap is compatible with the concept that PLAN forms a continuous age-related spectrum. Juvenile and adult presentations can overlap in terms of cerebellar atrophy, brain iron accumulation, cognitive decline, psychiatric symptoms, dystonia, and parkinsonism [20,21,26]. Consequently, rigid subtype labels may be less informative than a detailed description of age at onset, tempo of progression, predominant neurological systems involved, and neuroimaging findings.

From a diagnostic perspective, progressive loss of previously acquired language or motor abilities, particularly when accompanied by gait deterioration, hyporeflexia, contractures, optic abnormalities, or cerebellar atrophy should prompt reconsideration of an isolated neurodevelopmental diagnosis. MRI sequences sensitive to susceptibility effects and molecular testing that includes exon-level copy-number analysis are essential. When more than one potentially relevant variant is identified, segregation and phase analysis should be pursued whenever feasible.

This report has several additional limitations. First, because the family fled the war in Ukraine abruptly as refugees, they arrived in Poland without physical medical documentation, and their historical medical records were inaccessible. Consequently, our reconstruction of the clinical course relies substantially on maternal recall. Although the caregiver history was clinically coherent and plausible, and the major reported developmental changes were consistent with the subsequently documented juvenile PLAN-like phenotype, the absence of contemporaneous medical records remains a source of clinical uncertainty. Therefore, the exact timing and specific details of early symptom onset and milestones could not be independently verified. Standardized cognitive and behavioral assessments were not available. The copy-number gain was not confirmed by an orthogonal method and was not characterized at the breakpoint or transcript level; no functional studies were available. Finally, the small sample size, relatedness of the patients, and unresolved variant phase limit conclusions regarding variant pathogenicity and phenotypic prediction.

Despite these limitations, the concordant clinical, MRI, and ENG findings provide a clinically informative description of a juvenile PLAN-like phenotype in two siblings carrying p.Arg645Gln and an unresolved exon 4–7 copy-number gain. The findings are broadly concordant with previous reports involving p.Arg645Gln, but do not independently attribute the phenotype to this variant or establish a variant-specific phenotype. The cases also illustrate how unresolved phase can substantially constrain interpretation even when the phenotype is compelling.

## 4. Conclusions

Two siblings with childhood-onset regression, gait impairment, epilepsy or paroxysmal events, cerebellar atrophy, basal ganglia susceptibility changes, optic nerve thinning and selective motor axonal involvement had the same heterozygous *PLA2G6* p.Arg645Gln variant and exon 4–7 copy-number gain. Their phenotype is highly suggestive of juvenile PLAN. However, the absence of segregation analysis and structural characterization of the copy-number gain means that biallelic *PLA2G6* involvement and a definitive molecular diagnosis cannot be confirmed. The report underscores the importance of susceptibility-weighted MRI, exon-level copy-number analysis, orthogonal CNV confirmation, and determination of variant phase in children with progressive neurodevelopmental regression.

## Figures and Tables

**Table 1:** Clinical and diagnostic findings in the two siblings.

Feature	Patient 1	Patient 2
Sex and age	Male, 14 years old	Female, 9 years old
Early development	Normal	Normal
Symptom onset	3 years: nocturnal episodes of increased tone; 5 years: gait instability and speech regression.	2 years: gait deterioration; 3 years: speech regression and tonic seizures.
Initial diagnosis	Autism spectrum disorder	Epilepsy (treated with valproic acid)
Neurological examination	Nonverbal; increased muscle tone; ankle contractures, calf atrophy and scoliosis. Patellar and left Achilles reflexes absent; right Achilles reflex weak. Unable to walk independently; substantial support required.	Simple sentences; increased upper-limb muscle tone and ankle contractures. Patellar and Achilles reflexes absent. Broad-based unsteady gait requiring hand support.
Brain MRI	Symmetric cerebellar atrophy, SWI hypointensity of the globus pallidus and substantia nigra highly suggestive of iron accumulation, and bilateral optic nerve thinning.	
EEG	3 years: right-sided epileptiform discharges. Later EEG: abnormal spatial organization with suspected right frontal epileptiform activity. The later paroxysmal events were not confirmed as epileptic.	6 years: left temporal and right frontocentral sharp and slow waves. At admission: anterior theta slow waves.
ENG	Selective motor axonal involvement: reduced motor response amplitudes in both peroneal nerves. Mildly prolonged F-wave latencies, of nonspecific significance.	Mild selective motor axonal involvement: reduced peroneal motor response amplitudes recorded from extensor digitorum brevis, with relative sparing of tibialis anterior responses.
Laboratory findings	Mild CK elevation (393 U/L; reference 39–308 U/L) and lactate elevation (3.4 mmol/L; reference 0.5–2.2 mmol/L), of uncertain cause.	Low vitamin D, subtherapeutic valproate, mildly increased aspartate aminotransferase and thrombocytosis. CK and lactate within reference ranges.
*PLA2G6* findings	Heterozygous c.1934G>A (p.Arg645Gln) and a copy-number gain encompassing exons 4–7. Limitations in both siblings: unresolved phase, no orthogonal CNV confirmation, and no breakpoint or transcript-level characterization.	

## Data Availability

The original contributions presented in this study are included in the article. Further inquiries can be directed to the corresponding author.

## Abbreviations

ACMG	American College of Medical Genetics and Genomics
ANAD	atypical neuroaxonal dystrophy
CARE	CAse REport guidelines
CK	creatine kinase
ClinGen	Clinical Genome Resource
CNV	copy-number variant
DWI	diffusion-weighted imaging
EEG	electroencephalography
ENG	electroneurography
FLAIR	fluid-attenuated inversion recovery
GRCh38	Genome Reference Consortium human genome build 38
IMiD	Institute of Mother and Child
INAD	infantile neuroaxonal dystrophy
iPLA2β	calcium-independent phospholipase A2 beta
MANE	Matched Annotation from NCBI and EMBL-EBI
MLPA	multiplex ligation-dependent probe amplification
MRI	magnetic resonance imaging
mtDNA	mitochondrial DNA
NBIA	neurodegeneration with brain iron accumulation
NGS	next-generation sequencing
OMIM	Online Mendelian Inheritance in Man
PLAN	PLA2G6-associated neurodegeneration
QSM	quantitative susceptibility mapping
SWI	susceptibility-weighted imaging
